# Cytokine response and damages in the lungs of aging Syrian hamsters on a high-fat diet infected with the SARS-CoV-2 virus

**DOI:** 10.3389/fimmu.2023.1223086

**Published:** 2023-07-14

**Authors:** Gleb Fomin, Kairat Tabynov, Rinat Islamov, Nurkeldi Turebekov, Duman Yessimseit, Toktasyn Yerubaev

**Affiliations:** ^1^ Central Reference Laboratory, Aikimbayev’s National Scientific Center for Especially Dangerous Infections, Almaty, Kazakhstan; ^2^ Department of Biodiversity and Bioresources, Faculty of Biology and Biotechnology, Al-Farabi Kazakh National University, Almaty, Kazakhstan; ^3^ International Center for Vaccinology, Kazakh National Agrarian Research University, Almaty, Kazakhstan

**Keywords:** SARS-CoV-2, COVID-19, Syrian hamster, cytokine response, high-fat diet

## Abstract

Hypertriglyceridemia, obesity, and aging are among the key risk factors for severe COVID-19 with acute respiratory distress syndrome (ARDS). One of the main prognostic biomarkers of ARDS is the level of cytokines IL-6 and TNF-α in the blood. In our study, we modeled hyperglyceridemia and hypercholesterolemia on 18-month-old Syrian hamsters (*Mesocricetus auratus*). By 18 months, the animals showed such markers of aging as weight stabilization with a tendency to reduce it, polycystic liver disease, decreased motor activity, and foci of alopecia. The high-fat diet caused an increase in triglycerides and cholesterol, as well as fatty changes in the liver. On the third day after infection with the SARS-CoV-2 virus, animals showed a decrease in weight in the groups with a high-fat diet. In the lungs of males on both diets, there was an increase in the concentration of IFN-α, as well as IL-6 in both males and females, regardless of the type of diet. At the same time, the levels of TNF-α and IFN-γ did not change in infected animals. Morphological studies of the lungs of hamsters with SARS-CoV-2 showed the presence of a pathological process characteristic of ARDS. These included bronchointerstitial pneumonia and diffuse alveolar damages. These observations suggest that in aging hamsters, the immune response to pro-inflammatory cytokines may be delayed to a later period. Hypertriglyceridemia, age, and gender affect the severity of COVID-19. These results will help to understand the pathogenesis of COVID-19 associated with age, gender, and disorders of fat metabolism in humans.

## 1 Introduction

COVID-19 can manifest itself in both mild asymptomatic and severe fatal forms. The course of the disease is often complicated by various factors. These factors include immunosuppressive therapy, age-related changes, and most importantly, chronic diseases. It is known that patients from older age groups with comorbidities are more susceptible to the severe course of COVID-19 (1, 2).

The lungs are the main target organ of SARS-CoV-2. The virus causes acute respiratory distress syndrome (ARDS). This pathological condition usually involves an inflammatory process in the lungs with widespread diffuse alveolar involvement (3). Extrapulmonary lesions of the circulatory, nervous, hepatobiliary, and genitourinary systems are also observed with COVID-19 (4–6). It is also known that there is an increase in the concentration of some pro-inflammatory cytokines causing a condition called cytokine storm in patients with severe COVID-19 (1, 2, 7). One of the factors contributing to this condition is the metabolic syndrome. This syndrome is a cluster of pathological conditions that can manifest themselves in various combinations: obesity, insulin resistance syndrome, arterial hypertension, and atherogenic dyslipidemia (8). High triglyceride levels are known to be associated with severe COVID-19 (9, 10). Given the above, we can say that the combination of age-related and metabolic changes in the body plays a significant role in the severity of COVID-19.

Due to the need to study the pathogenesis of COVID-19 in comorbid conditions, we chose the Syrian hamster (*Mesocricetus auratus*). The choice is based on the fact that the animals show clinical signs of COVID-19 when infected with SARS-CoV-2. We used animals belonging to the aging age group (18 months) that were on a high-fat diet. As a result, we obtained a model of laboratory animals with hypercholesterolemia, hypertriglyceridemia, and fatty changes in the liver. In this study, we demonstrate how metabolic disorders, age, and gender affect pathological changes in the lungs of Syrian hamsters infected with SARS-Cov-2.

## 2 Materials and methods

### 2.1 Animal keeping

The study used outbred Syrian hamsters (*Mesocricetus auratus*) of both genders, 18 months old. All animals were kept in the 3rd Animal Biosafety Level (ABSL-3) facility of the Central Reference Laboratory (CRL) of the Aikimbayev’s National Scientific Center for Especially Dangerous Infections (Almaty, Republic of Kazakhstan), hereinafter referred to as NSCEDI. The animals were kept in individually ventilated cages (IVC) with a HEPA filter (Allentown, USA) on a special autoclavable ssniff^®^ bedding (Spezialdaten GmbH, Germany). The conditions corresponded to generally accepted standards: ambient temperature 21 ± 2°C, humidity 50 ± 10%, and a day/night cycle (10:14). The diet included a standardized complete ssniff^®^ feed for hamsters (cat. No. V 2144-000) (Spezialdaten GmbH, Germany). Water and feed *ad libitum*. The animals were weighed using Adventurer RV 1502 scales (Ohaus, China). The study protocol was approved by IACUC No. 14.

### 2.2 Pathogen

The study used the strain SARS-CoV-2, isolated earlier (11, 12). The complete sequence of the protein was published in the GISAID database under the number EPI_ISL_514093. The virus was cultured on a monolayer Vero E6 cell line in Dulbecco’s Modified Eagle Medium (DMEM) with added 2% bovine fetal serum and antimycotic antibiotic solution (penicillin 10,000 U, streptomycin 10 mg. and amphotericin B 25 μg) (Gibco, USA) at a temperature 37°C in a CO_2_-incubator in an atmosphere with 5% carbon dioxide for three days. One of the infected hamsters did not survive the anesthesia.

### 2.3 Study design

The experimental animals were randomized into 8 groups consisting of 6 individuals (**Table 1**). Half of the animals were kept on a standard diet (RD), and the other half from 14 to 18 months received a high-fat diet (HF) with 20% coconut oil and 0.2% ssniff^®^ cholesterol for hamsters (cat. no. E21413-3402) (Spezialdaten GmbH, Germany). Under anesthesia, blood was taken from the gingival vein to assess biochemical changes in the blood of hamsters before and after feeding diets.

**Table 1:** Distribution of animals into groups.

Diet	Control		Infected with SARS-CoV-2	
	Male	Female	Male	Female
**Standard (RD)**	6	6	6	6
**High-fat (HF)**	6	6	6	6
**Total**	12	12	12	12

Experimental animals were infected by the intranasal route. For this, hamsters were anesthetized by intraperitoneal injection of solutions of ketamine and xylazine at doses of 100 and 10 mg/kg, respectively. Then, 10 μl of DMEM containing the hCoV-19 virus at a dose of 10^6.0^ TCID_50_ was added to each nostril. A virus-free nutrient medium was injected into the nostrils of the control animals.

The animals were weighed on days 0, 1, 2, and 3 post-infection (dpi). After that, the animals were euthanized and samples of lung and liver tissue were collected for ELISA and histological studies.

### 2.4 Biochemical analysis

Serum was isolated from the blood of the hamsters by centrifugation at 6000 rpm for 15 minutes for clinical chemistry testing. Serum samples were examined on the A-25 biochemical analyzer (Biosystem, Spain). The following parameters were measured: alanine aminotransferase (ALT), aspartate aminotransferase (AST), glucose (Glu), cholesterol (Chol), and triglycerides (Trgly).

### 2.5 ELISA test of the cytokine level in the lungs

To study the level of cytokines in the lungs, the tissue was homogenized in a ratio of 1 to 10 PBS (Sigma, USA) with the addition of Protease Inhibitor A32965 (Thermo Scientific, USA). Cytokines were measured quantitatively by using the ELISA sandwich method with hamster kits from MyBioSource (USA) for TNF- α (MBS7606475), IL-6 (MBS700950), IFN-α (MBS010919), and IFN-γ (MBS010146)

### 2.6 Histological processing of tissues

The organs were fixed in a 10% formalin solution. After 24-hour fixation the organs were subjected to histological processing which involved four portions of isopropanol (BioVitrum), and two portions of the clearing agent Bio-Clear (Bio-Optica, Italy). Then the material was soaked in four portions of paraffin medium Histomix (BioVitrum) with the subsequent embedding of histological blocks. After that, the blocks were cut on a semi-automatic microtome MZP-01 (KB Technom), as a result of which sections were obtained with a thickness of 5 micrometers (μ). Then the sections were dewaxed in two portions of xylene and three portions of ethanol, and after that, they were stained with Mayer’s hemalum (BioVitrum) and aqueous 1% eosin (DiaPath). This was followed by a clarification in ethanol and two portions of Bio-Clear. In the end, the sections were closed with cover glasses using a synthetic medium Bio Mount (Bio-Optica, Italy). The specimens were studied using the Mshot microscope (China), MF52-N model. The photographs were taken with a Mshot MS23 camera (China) in the MShot image analysis system (China). Each slide was quantified based on the severity of histologic changes, including interstitial pneumonia, alveolitis, bronchiolitis, alveolar destruction, interstitial infiltration, pulmonary hemorrhage, and peribronchiolar inflammation. Scoring of pathological changes in the lung sections: 4 points = extremely severe; 3 points = severe; 2 points = moderate; 1 point = mild; 0 points = no changes (13).

### 2.7 Biosafety

All works were carried out in ISO 35001:2019 certified laboratories of the 3rd biosafety level (BSL3) in the CRL of NSCEDI. The works were carried out in a biosafety cabinet SG404, class II, type A2 (Baker, USA). A biosafety specialist of the CRL participated in all works.

### 2.8 Statistical data analysis

All quantitative results are presented as the average ± SD. Statistical significance was assessed by parametric and nonparametric criteria. The body weight dynamics and cytokine level were assessed by a one-way ANOVA with Tukey’s multiple comparisons test. Biochemical analysis was assessed using the unpaired Mann-Whitney test. Histological scoring was assessed by the Kruskal-Wallis test with Dunn’s multiple comparisons tests. The differences were considered significant for the p values less than 0.05. Statistical tests were conducted using GraphPad Prism version 9.0.0 (GraphPad Software, La Jolla, CA, USA).

## 3 Results

### 3.1 Modeling of hypercholesterolemia and hyperglyceridemia in hamsters

**Figure 1:**
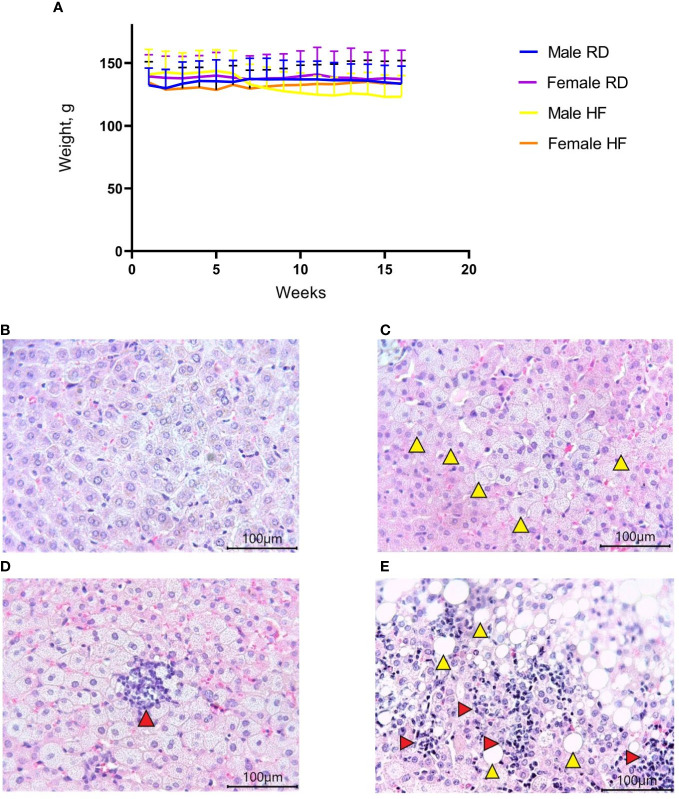
Body weight change and liver histology of the Syrian hamster on a high-fat diet. **(A)** Weight dynamics analysis within four months. **(B)** Liver parenchyma in animals on the RD diet (x400 magnification). **(C)** In hamsters on the HF diet, foci with microvesicular fatty changes in hepatocytes (yellow arrowheads) were observed (x400 magnification); **(D)** low-grade inflammatory infiltrate (red arrow) in the area of microvesicular fatty changes (x400 magnification). **(E)** moderate inflammatory infiltrate (red arrowheads) in the area of macrovesicular changes (yellow arrowheads) (x400 magnification). All histological images with Hematoxylin-Eosin staining.

Hamsters are a suitable model for diet-induced hypercholesterolemia, hypertriglyceridemia, and obesity (14). For the development of hypercholesterolemia and hypertriglyceridemia, the animals received a high-fat diet with added cholesterol for four months. There was a decrease in weight in males on the HF diet (p<0.001). At the same time, a slight increase in weight was noted in females from the HF group (p<0.001) (**Figure 1A**). Histological examination of the liver showed the presence of fatty changes, with inflammatory infiltrates (**Figures 1C–E**), compared with the standard diet group (**Figure 1B**).

**Figure 2:**
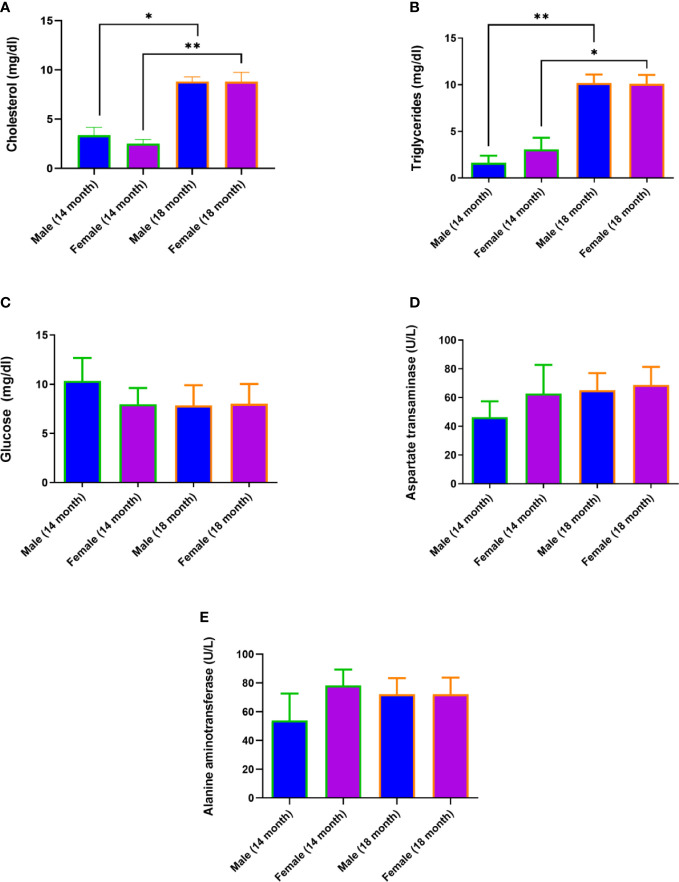
Diet-induced hypercholesterolemia and hypertriglyceridemia in Syrian hamsters. Biochemical study of blood serum showed an increase in cholesterol **(A)** and triglycerides **(B)** in 18-month-old animals. Differences in glucose **(C)**, aspartate aminotransferase **(D)**, and alanine aminotransferase **(E)** levels were not observed. n = 5 animals per group. P-value was derived using the Mann-Whitney test. Asterisks denote the level of significance observed, as follows: *p ≤ 0.05; **p ≤ 0.01.

An increase in cholesterol levels was observed in 18 females (95% confidence interval (CI), 7,6-10,0) compared to 14-month-old females (95% CI, 2,0-3,0; p<0.01) and in 18-month-old males (95% CI, 8,2-9,4) compared to 14-month-old males (95% CI, 2,4-4,4; p<0.05) (**Figures 2A, B**). An increase in triglyceride levels was observed in 18 females (95% CI, 8,9-11,3) compared to 14-month-old females (95% CI, 1,5-4,6) p<0.05) and in 18-month-old males (95% CI, 9,1-11,3) compared to 14-month-old males (95% CI, 0,7-2,6; p<0.01) (**Figures 2A, B**). At the same time, glucose levels, aspartate aminotransferase, and alanine aminotransferase did not change (**Figures 2C–E**).

### 3.2 Hamsters aging

**Figure 3:**
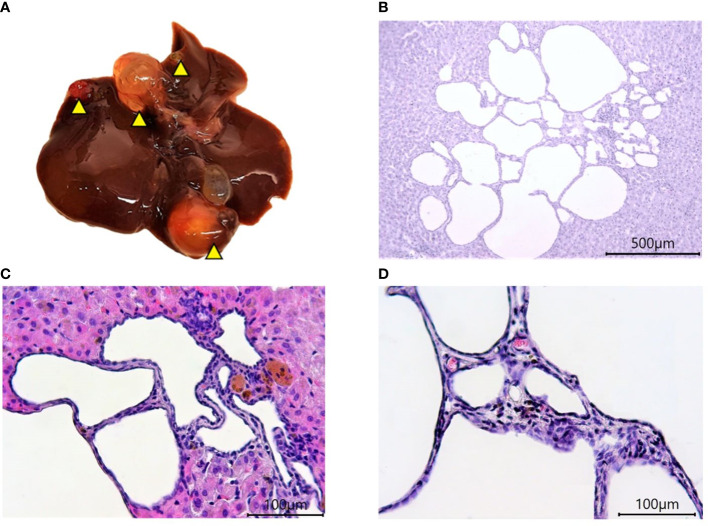
Polycystic liver disease in 18-month-old Syrian hamsters. **(A)** Multi-lobular cysts in the liver (yellow arrowheads). **(B)** large multi-locular cysts (x40 magnification),. **(C)** small cysts (x100 magnification). **(D)** Cyst walls (x400 magnification). All histological images with Hematoxylin-Eosin staining.

The old age of hamsters begins at 18 months and is characterized by a number of significant changes in the body. These include degenerative processes in the gonads (15), polycystic liver disease, and other characteristic signs (16). Signs of aging in experimental animals were established during the external examination, after the autopsy, and macroscopic and histological examination of organs. Motor activity of hamsters decreased and foci of alopecia appeared, which became larger over time. At autopsy, most of the individuals were found to have characteristic multi-lobular mono- and multi-locular cysts of various sizes (**Figure 3**).

### 3.3 Infection of hamsters with SARS-CoV-2

**Figure 4:**
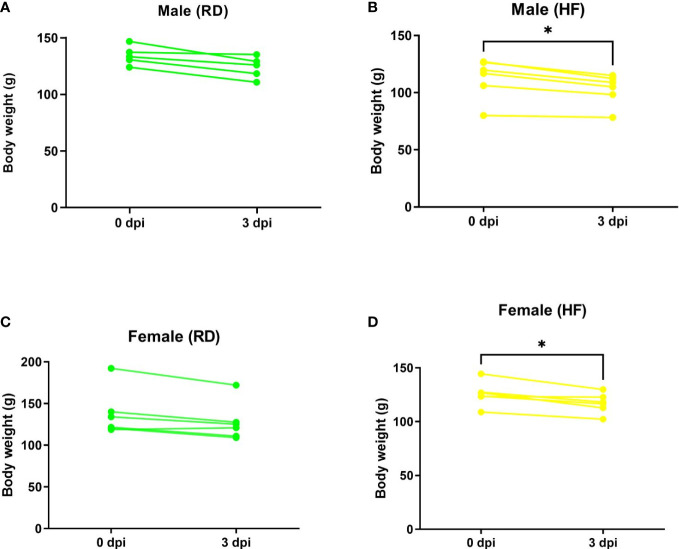
Weight dynamics of infected hamsters. Animals on a normal diet **(A–C)** did not show significant weight loss. Weight was significantly reduced in males **(B)** and females **(D)** on a high-fat diet. P-value was derived using one-way ANOVA with Tukey’s multiple comparisons test. Asterisks denote the level of significance observed, as follows: *p ≤ 0.05.

After infection, animals fed both standard RD and high-fat HF diets showed daily clinical signs of COVID-19, such as behavior and body weight dynamics. Weight loss was noted on day 3 after infection (**Figure 4**).

**Figure 5:**
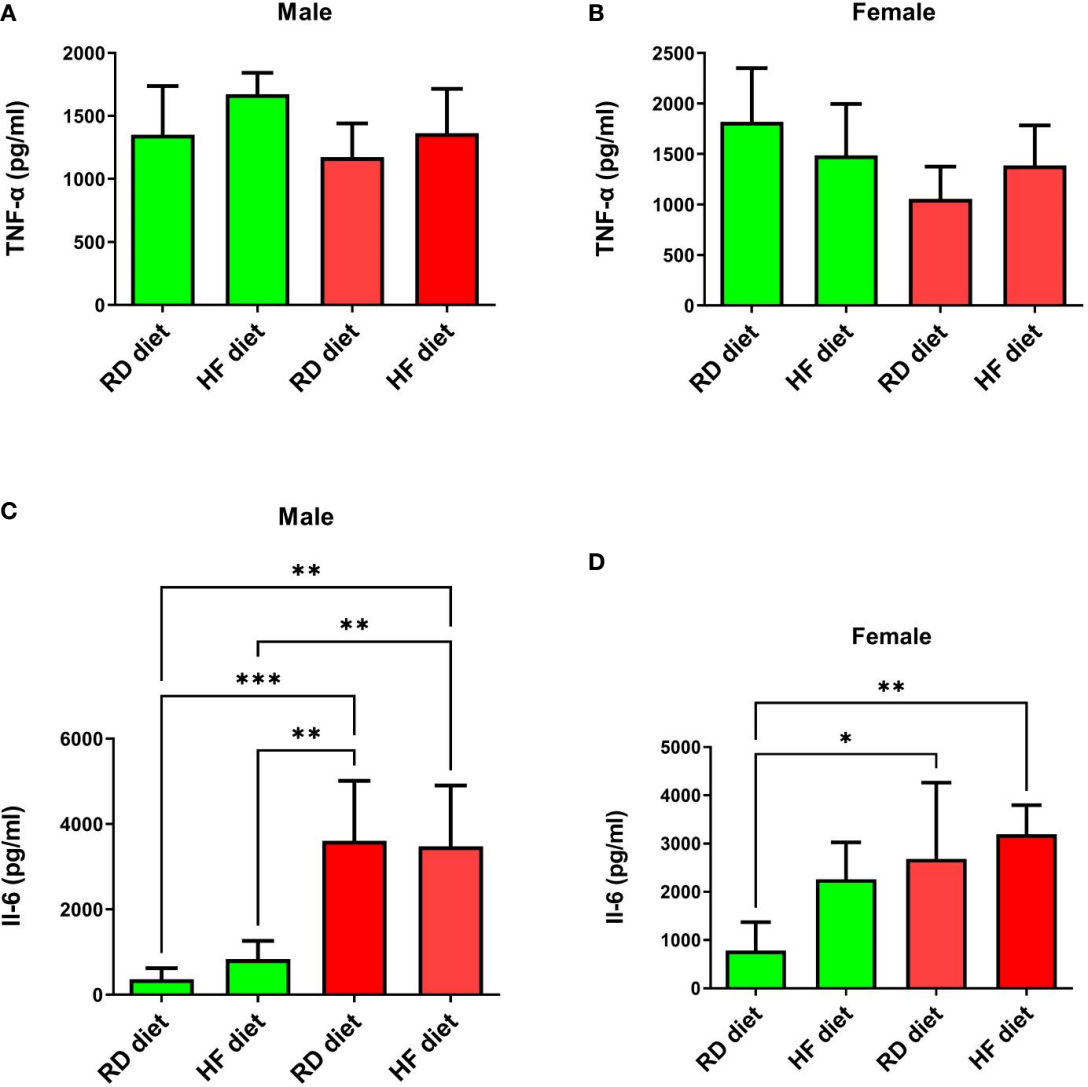
Cytokine response of TNF-α and Il-6 in the lungs of Syrian hamsters on different diets infected with SARS-CoV-2. No changes were found in the level of TNF-α **(A, B)**. And IL-6 levels increased in infected animals **(C, D)**. Cytokine levels were measured in lung homogenates from euthanized animals. n = 5 animals per group. Green bar – healthy animals; Red bar - animals infected with SARS-CoV-2. P-value was derived using one-way ANOVA with Tukey’s multiple comparisons tests. Asterisks denote the level of significance observed, as follows: *p ≤ 0.05; **p ≤ 0.01; ***p ≤ 0.001.

Next, the animals were euthanized and lungs were collected to determine the levels of TNF-α, IL-6, IFN-α, and IFN-γ. The lungs and liver were also histologically examined. Changes in the levels of TNF-α in the lungs of all groups were not observed (**Figures 5A, B**). There was also a significant increase in IL-6 levels in males from the infected groups, both on the RD diet (95% CI, 1867,0-5351,0) and the HF diet (95% CI, 1705,0-5247,0), compared with healthy animals on the RD diet (95% CI, 45,3-682,5; p<0.001 and p<0.01 respectively), and the HF diet (95% CI, 289,9-1369,0; p<0.01, both) (**Figure 5С**). Infected females on the RD (95% CI, 721,1-4647,0) and HF diets (95% CI, 2447,0-3945,0) showed an increase in IL-6 compared to healthy females on the RD diet (95% CI, 41,3-1519,0; p<0.05 and p<0.01, respectively) (**Figure 5D**).

**Figure 6:**
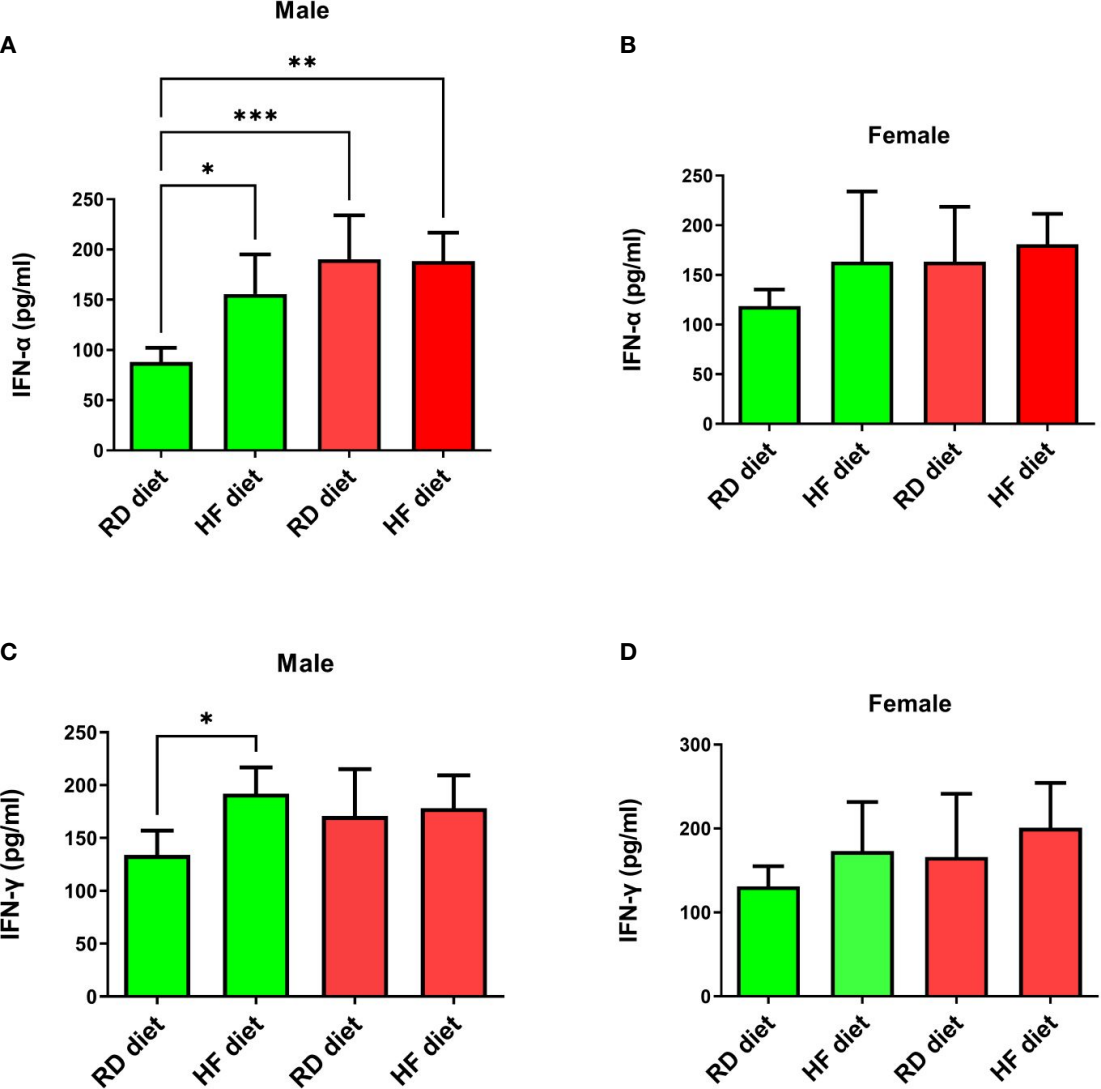
Cytokine response of IFN-α and IFN-γ in the lungs of Syrian hamsters on different diets infected with SARS-CoV-2. Сhanges in interferon levels were noted in males (**A–C**), but not in females (**C, D**). Cytokine levels were measured in lung homogenates from euthanized animals. n = 5 animals per group. Green bar – healthy animals; Red bar - animals infected with SARS-CoV-2. P-value was derived using one-way ANOVA with Tukey’s multiple comparisons tests. Asterisks denote the level of significance observed, as follows: *p ≤ 0.05; **p ≤ 0.01; ***p ≤ 0.001.

The study of the effect of the SARS-CoV-2 virus on type I and II interferons showed the following results. IFN-α levels significantly increased in SARS-CoV-2-infected males on RD (95% CI, 135,9-244,7) and HF diets (95% CI, 153,1-223,7) compared to healthy animals on RD diet (95% CI, 70,3-105,6; p<0.001 and p<0.01 respectively) (**Figure 6A**). At the same time, an increase in the production of this cytokine was observed in the group of healthy males on the HF diet (95% CI, 106,9-204,5) in comparison with healthy males on the RD diet (95% CI, 70,27-105,6; p<0.05). However, changes in the levels of IFN-α in the lungs of females were not observed (**Figure 6B**). Another key cytokine of inflammation, IFN-γ, did not respond in either males or females (**Figures 6C, D**). Only a difference was observed among healthy males between HF (95% CI, 161,1-222,8) and RD diets (95% CI, 105,2-162,5; p<0.05).

**Figure 7:**
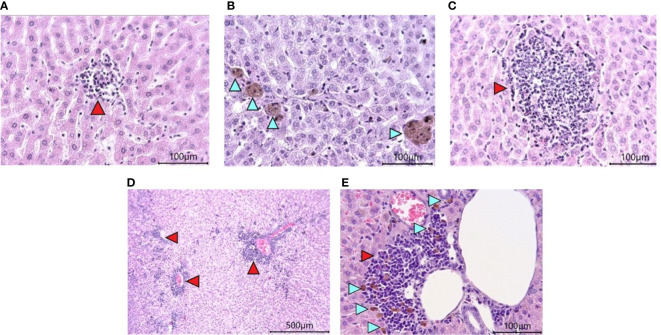
Histological examination of the liver of Syrian hamsters. **(A)** low-grade mononuclear infiltrate in the hamster liver parenchyma on the RD diet (x400 magnification). **(B)** pigment in hamster hepatocytes on the RD diet (blue arrowheads, x400 magnification). **(C)** - moderately marked granulocyte infiltrate with predominant neutrophils in the liver parenchyma of a hamster on the RD diet infected with SARS-CoV-2 (x400 magnification); **(D)** Marked multifocal infiltrates (red arrowheads) (x100 magnification) with **(E)** pigmented macrophages (blue arrowheads) (x400 magnification) in the liver triad region of a hamster infected with SARS-COV-2. n = 6 animals per group. All histological images with Hematoxylin-Eosin staining.

Histological examination of the liver revealed the presence of pathological changes of varying degrees within the same group. A common characteristic for all animals, regardless of the group, was the presence of dilated sinusoidal spaces and multifocal foci of low-grade mononuclear-macrophage infiltration in the parenchyma (**Figure 7A**). Single foci were encountered with a group of pigment-filled hepatocytes (**Figure 7B**). In the groups infected with SARS-CoV-2, there were marked foci of granulocyte infiltration (**Figure 7C**), as well as multifocal lymphocytic foci of inflammation of the liver triad region (**Figures 7D, E**). Some of them had pigment-filled macrophages (**Figure 7E**).

**Figure 8:**
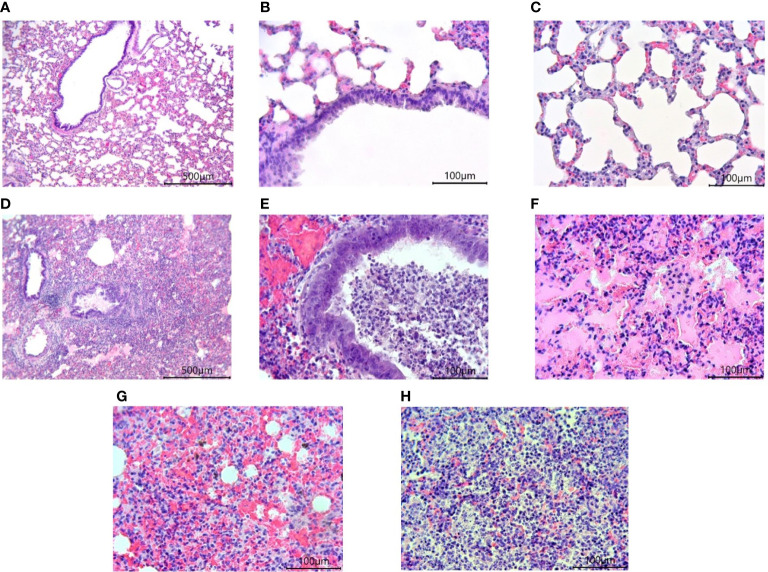
Histological examination of the lungs of Syrian hamsters infected with SARS-CoV-2. **(A–C)** lungs in healthy animals (x100 magnification -A) (x400 magnification **B, C**; **(D–H)** lungs in infected animals: **(D)** the focus of bronchointerstitial pneumonia (x400 magnification); **(E)** peribronchial infiltrates and cell debris in the bronchi lumen (x400 magnification); **(F)** edema and destruction of the pulmonary alveoli (x400 magnification); **(G)** hemorrhages and destruction of pulmonary alveoli (x400 magnification); **(H)** intra-alveolar infiltrates and cell debris (x400 magnification). n = 6 animals per group. All histological images with Hematoxylin-Eosin staining.

**Figure 9:**
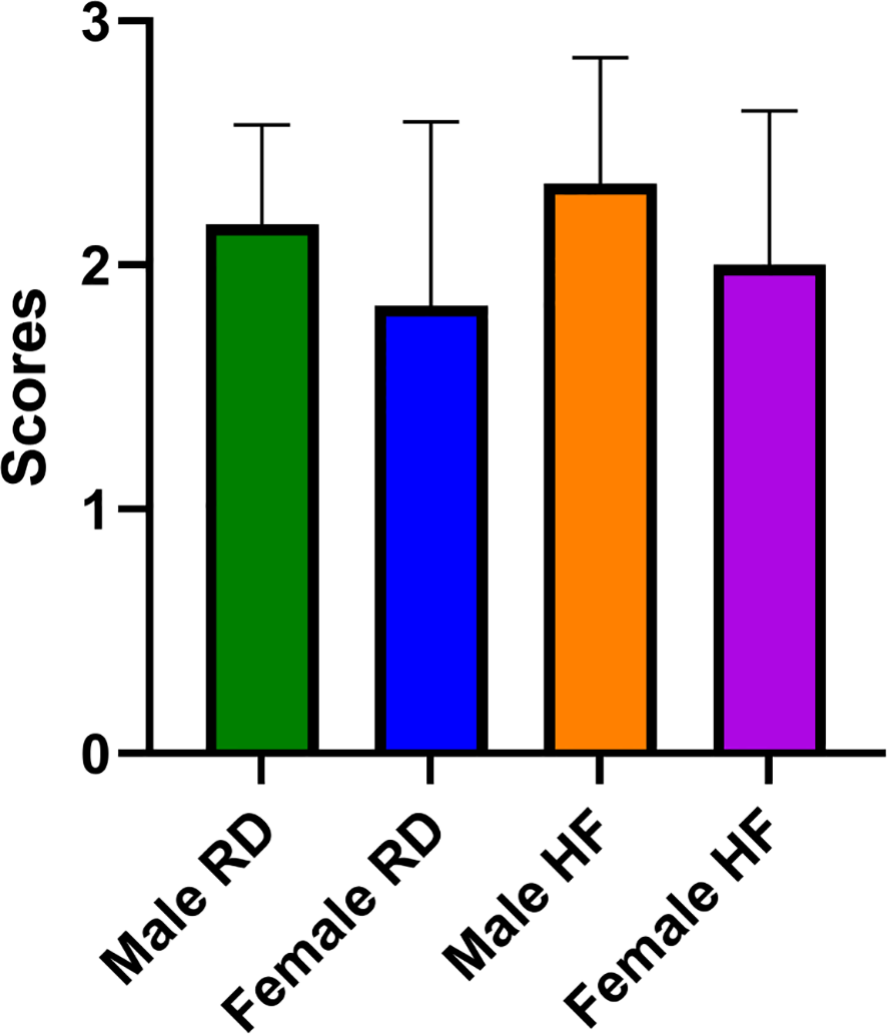
Scoring of pathological changes in the lungs of SARS-CoV-2 infected animals. There are no significant differences were found between groups of infected animals. A 4-point scale was used: 4 points = extremely severe; 3 points = severe; 2 points = moderate; 1 point = mild; 0 points = no changes. P-value was derived using the Kruskal-Wallis test with Dunn’s multiple comparisons tests.

Morphological studies of the lungs of hamsters infected with the SARS-CoV-2 virus showed the presence of a pathological process characteristic of ARDS in the form of bronchointerstitial pneumonia and diffuse alveolar damages (**Figure 8**). Extensive foci of changes coexisted with healthy sites within the same group, and varied in severity from group to group, but were similar (**Figure 9**). Pathological changes represented significant peribronchial infiltrates and desquamation of the epithelium in the bronchi lumen. Edema, interstitial and intra-alveolar infiltrates were observed in the lung parenchyma, in which lymphocytes and single neutrophils predominated. Part of the alveolar-capillary units in the foci of edema, hemorrhage, and cell debris was also disturbed.

## 4 Discussion

The study of COVID-19 pathogenesis in hamsters of different ages has been carried out repeatedly. Differences in the weight loss dynamics and the development of lung lesions in 6-week-old and 34-week-old hamsters were shown (17). The response of adaptive immunity expressed in the production of neutralizing antibodies was reduced in aging hamsters (18). In addition, the pathogenesis of COVID-19 in young hamsters on the High-Fat High-Sugar diet was studied. Researchers have found more pronounced pathological processes in hamsters on the High-Fat High-Sugar diet, as well as an increase in the level of IFN-γ in the blood (19).

Our study illustrated a COVID-19 model in aging 18-month-old Syrian hamsters with hypercholesterolemia and hyperglyceridemia. Stabilization of the weight of animals from 14 to 18 months can indicate the onset of aging (20). Evidence of aging was also alopecia foci and polycystic liver disease, characteristic of individuals older than one year (21). The formation of cysts in the liver in hamsters is associated with hyperplasia of the bile ducts and does not refer to neoplastic changes (22). Signs of obesity appeared without a significant weight change or an increase in the amount of adipose tissue. But at the same time, micro-vesicular fatty changes in the liver and an increase in the level of triglycerides and cholesterol in animals on the HF diet were noted. This condition is similar to the type of obesity with normal weight (Metabolically Obese Normal Weight, MONW), in which the body mass index remains normal, but metabolic changes are noted, manifested in pathological changes in the liver and dyslipidemia (23, 24).

Infected animals showed signs characteristic of COVID-19, and first of all, a decrease in body weight (17). This clinical sign can be considered the most characteristic and can be used as one of the markers of the pathogenesis of COVID-19. Although the mRNA expression of a large number of cytokines in the lungs of hamsters infected with SARS-CoV-2 has been well studied (25), however, the level of cytokines measured by ELISA in the blood and other organs differs (19, 26). In clinical practice, measurement of the cytokine level in the lungs is usually impossible. However, these parameters are important for understanding the pathogenesis of COVID-19 at the tissue level. One of the main pleiotropic cytokines that respond to viral tissue damage is TNF-α (27). This cytokine level rises in the blood and lungs of young hamsters infected with SARS-CoV-2 (28). Also, the level of expression of mRNA TNF-α in the spleen on 2 dpi is quite high but then decreases rapidly (29). Cytokine IL-6 is among the prognostic biomarkers of severe COVID-19 (22). However, in experimental studies of COVID-19, the production of IL-6 in blood serum and bronchoalveolar lavage fluid of hamsters was not shown (26, 28). Whereas, the expression of mRNA IL-6 increases (30). Pronounced signs of ARDS were found in the lungs of hamsters, which did not have significant pathomorphological differences between groups with different diets. Although, the level of IL-6 in the lungs was increased only in males. The observed lesions could occur through activation of the matrix metalloproteinase-9 (MMP-9) macrophages (30). The different sensitivity of males and females to SARS-CoV-2 has previously been shown for other parameters (31, 32). The difference in IL-6 levels in blood and tissues could be due to the fact that it regulates the response of CD4+ T cells, which produce a number of cytokines that damage the lungs (33). In general, gender differences affect the response of the IL-6 cytokine differently.

Type I interferons, which include IFN-α, play a significant role in antiviral immunity (34). Its early concentrations in the plasma of patients with COVID-19 are positively correlated with the disease duration. The ability of IFN-α to interfere with the transcription of T-regulatory cells and reduce their number in obesity is known, which can explain the absence of significant changes in the levels of IFN-γ (25). However, the increase in IFN-α production during COVID-19 can also have a downside. IFN-α enhances the hyperinflammatory response in the lungs caused by pro-inflammatory cytokines (35). At the same time, we should note the absence of an immune response from IFN-γ in all animals and TNF-α in females, which is associated with lung inflammatory cell death (36). One of the likely reasons for this phenomenon could be a decrease in the immune response as a result of “inflammageing” (37). This problem is associated with a decrease in the expression of Toll-like receptors (TLRs) responsible for recognizing pathogens. It is also possible that the activity of IFN-γ genes in the lungs is shifted to a later date of infection and this is due to the age of the animals (38).

## 5 Conclusion

This study showed that modeling obesity in aging 18-month-old hamsters using a high-fat diet with cholesterol does not lead to weight gain in animals. However, lipid metabolism disorders develop with the accumulation of fat in the liver. When modeling COVID-19 in hamsters, both on a standard and a high-fat diet, there were no significant differences in the pathological processes caused by the SARS-CoV-2 virus, except for weight, which decreased in groups on the high-fat diet. However, differences in the immune response for the key cytokine IFN-α in males and females have been shown. At the same time, IL-6 levels increased in both males and females. Summarizing the results, it can be assumed that the response of aging female hamsters to infection with the SARS-CoV-2 virus is weaker in the first days of infection.

## Data availability statement

The original contributions presented in the study are included in the article/**Supplementary Material**. Further inquiries can be directed to the corresponding author.

## Ethics statement

The study protocol was approved by IACUC No. 14 by the Ethics Committee of the Aikimbayev’s National Scientific Center for Especially Dangerous Infections.

## Author contributions

Equal contribution: GF, KT, and RI. First authorship: GF, KT, and RI. Senior authorship: KT and RI. Last authorship: NT, DY, and TY. Equal contribution and first authorship: GF. Equal contribution and senior authorship: KT and RI. Equal contribution and last authorship: NT, DY, and TY. All authors contributed to the article and approved the submitted version.
