# Supplementary material for: Cytokine response and damages in the lungs of aging Syrian hamsters on a high-fat diet infected with the SARS-CoV-2 virus
Source: Front Immunol. 2023 Jul 14;14:1223086. doi: 10.3389/fimmu.2023.1223086 (PMC10375707; doi:10.3389/fimmu.2023.1223086)
Supplement: Supplementary file 1 [file DataSheet_1.zip › S 1 Table..pdf]

S 1 Table. Weight dynamics of male Syrian hamsters during the experiment

**Males on RD diet (g)**												
**Weeks**	№1	№2	№3	№4	№5	№6	№7	№8	№9	№10	№11	№12
1	114,4	120,8	128,1	120,8	111,8	121,8	145,1	151,1	150,2	148,6	134,4	120,5
2	114,6	122,1	124,0	115,1	106,0	117,2	142,2	149,7	148,8	147,2	134,6	118,9
3	113,3	120,9	120,1	118,2	110,0	123,3	149,6	159,7	155,5	150,5	143,2	119,4
4	115,1	121,5	126,1	120,1	108,4	123,1	149,9	160,7	155,1	151,8	145,9	121,4
5	114,4	121,9	119,1	119,5	109,5	122,6	148,5	160,7	154,6	156,3	149,7	119,3
6	115,4	122,7	121,5	119,4	112,4	122,4	145,1	157,7	154,3	156,2	152,4	120,0
7	148,2	124,1	133,9	129,9	146,2	160,5	129,2	108,0	156,8	157,3	163,2	121,9
8	140,1	121,9	131,0	131,5	141,9	158,2	128,7	107,4	158,4	160,4	159,3	125,4
9	138,3	124,8	133,4	129,1	147,0	155,1	126,0	109,4	154,4	156,6	156,6	124,3
10	138,1	123,3	134,5	131,0	146,2	154,6	127,3	113,0	153,5	158,2	152,2	121,8
11	137,9	124,4	131,5	133,6	146,6	151,9	124,7	112,0	150,0	159,5	149,1	122,6
12	139,2	122,6	130,6	131,6	146,4	152,1	126,1	112,4	149,3	159,2	142,9	123,0
13	140,2	123,4	130,6	129,8	146,1	150,8	126,1	120,4	151,2	158,4	136,9	122,2
14	138,2	126,0	130,7	129,6	147,0	150,2	127,1	118,8	151,0	160,3	128,6	124,6
15	138,0	128,0	132,3	129,4	139,3	149,4	126,0	119,3	150,1	162,0	121,2	120,5
16	139,8	127,7	130,9	126,4	138,7	149,3	127,3	115,5	146,4	162,0	117,7	119,4
**Males on HF diet (g)**												
**Weeks**	№1	№2	№3	№4	№5	№6	№7	№8	№9	№10	№11	№12
1	109,7	152,6	158,1	146,8	132,5	120,6	127,4	143,8	129,6	150,9	133,4	186,0
2	108,6	160,3	163,9	154,7	134,0	121,7	132,7	147,8	137,3	162,1	143,4	145,0
3	113,4	161,3	166,7	153,6	131,3	121,9	129,5	150,1	133,6	157,8	137,2	141,8
4	113,9	162,4	169,5	159,4	130,3	121,9	130,8	147,7	132,7	158	137,2	146,3
5	117,2	164,5	166,4	160,2	131,3	122,7	130,5	149,1	133,7	160,4	142,4	146,3
6	116,1	168,7	166,0	161,2	130,1	121,3	126,3	149,4	126,8	155,7	140,1	140,2
7	121,0	119,9	141,1	149,9	152,2	111,4	121,1	122,8	113,0	146,6	146,7	150,5
8	123,7	115,3	138,7	148,6	153,6	111,4	120,8	121,7	117,1	108,9	142,6	155,7
9	121,2	118,1	132,0	148,3	142,8	111,4	120,3	125,5	118	102,7	140,4	151,1
10	122,9	106,5	132,9	147,2	139,9	111,6	120,2	123,4	117,0	101,3	139,0	151,3
11	121,4	108,2	129,3	138,0	140,6	110,9	116,9	125,4	117,7	101,7	134,7	152,7
12	121,4	112,2	127,2	133,8	140,3	111,6	120,2	126,0	116,4	97,3	131,3	152,3
13	120,1	113,5	129,5	141,9	140,5	112,4	126,1	128,4	117,7	93,8	130,6	154,8
14	114,8	109,6	127,3	146,1	138,0	114,2	123,4	131,4	118,3	90,6	132,0	155,4
15	114,1	107,5	126,6	144,3	132,5	113,0	123,0	127,4	116,8	87,1	128,8	154,6
16	113,3	113,1	125,3	143,8	132,0	115,0	120,1	128,0	116,4	86,9	129,2	154,1
