# Supplementary material for: Cytokine response and damages in the lungs of aging Syrian hamsters on a high-fat diet infected with the SARS-CoV-2 virus
Source: Front Immunol. 2023 Jul 14;14:1223086. doi: 10.3389/fimmu.2023.1223086 (PMC10375707; doi:10.3389/fimmu.2023.1223086)
Supplement: Supplementary file 1 [file DataSheet_1.zip › S 2 Table..pdf]

S 2 Table. Weight dynamics of female Syrian hamsters during the experiment

Females on RD diet (g)												
**Weeks**	№1	№2	№3	№4	№5	№6	№7	№8	№9	№10	№11	№12
1	121,9	127,2	136,9	154,1	129,5	132,5	125,5	185,1	132,9	133,9	145,5	146,9
2	117,4	125,3	133,0	152,6	129,7	132,8	122,0	182,0	134,4	136,7	146,5	146,3
3	120,9	123,2	133,3	151,7	129,5	134,6	125,7	185,2	134,2	133,6	147,3	135,5
4	120,7	129,6	132,7	149,4	131,0	134,4	129,3	186,5	134,2	133,4	141,3	146,2
5	118,2	128,3	132,8	151,0	126,3	136,4	130,2	189,0	137,9	132,9	146,2	151,4
6	118,4	125,4	136,4	153,9	128,3	139,4	128,0	164,1	138,3	130,6	144,7	153,7
7	116,0	120,3	132,3	147,9	126,6	129,7	124,1	183,9	136,1	129,3	142,9	155,2
8	118,3	124,3	134,7	144,3	124,3	129,0	123,9	186,5	137,3	128,5	143,0	159,0
9	118,5	124,2	135,0	147,3	125,5	131,7	126,8	189,0	134,8	126,3	141,7	155,6
10	118,2	124,0	137,1	149,0	125,6	133,2	128,8	192,3	142,0	124,7	143,0	156,3
11	118,9	125,6	138,8	148,8	124,9	133,3	129,3	198,5	144,3	125,1	143,5	160,0
12	119,5	124,2	140,7	147,6	123,9	132,9	129,3	195,5	142,2	119,5	133,6	152,6
13	118,6	127,2	139,8	145,2	124,1	132,8	133,1	201,2	144,0	124,5	110,2	158,1
14	120,0	127,4	138,5	148,9	122,6	133,5	126,3	194,7	139,5	122,2	115,3	154,5
15	122,5	126,2	138,8	148,5	124,9	136,0	133,0	197,3	141,9	121,1	111,7	152,0
16	121,9	121,5	137,7	151,1	123,2	136,5	128,1	198,2	149,0	122,0	198,4	146,2
**Females on HF diet (g)**												
**Weeks**	№1	№2	№3	№4	№5	№6	№7	№8	№9	№10	№11	№12
1	122,8	161,3	134,4	155,8	147,0	139,7	122,1	140,2	119,3	142,9	124,6	104,1
2	117,7	157,6	131,3	149,0	140,4	131,7	116,8	132,8	113,8	134,5	121,4	99,5
3	110,8	158,7	131,7	149,6	142,2	132,9	119,9	137,2	115,2	135,9	122,3	100,7
4	114,8	156,4	130,2	150,4	143,2	132,8	120,9	140,4	115,5	136,9	123,6	102,2
5	118,2	157,5	128,9	147,5	125,5	131,0	119,9	136,0	116,2	136,4	124,0	103,0
6	118,2	157,2	124,8	149,3	147,0	135,1	124,6	143,2	120,4	140,1	127,2	104,9
7	120,2	149,2	127,2	149,3	146,7	125,2	125,8	139,1	114,2	133,3	120,0	104,3
8	123,0	150,8	129,6	151,1	144,6	130,3	127,4	140,1	116,8	136,5	120,9	106,7
9	124,8	152,0	132,2	148,7	145,8	130,2	127,8	139,2	119,8	139,8	117,8	109,9
10	127,8	159,1	134,5	149,5	145,0	130,6	131,0	134,4	116,9	141,2	112,5	105,8
11	129,3	162,2	133,5	151,9	142,6	135,0	132,0	126,4	118,1	142,8	111,9	112,5
12	128,4	159,7	135,8	153,4	146,8	134,3	129,7	119,0	119,7	145,1	111,6	113,2
13	129,6	163,9	135,2	155,0	148,8	134,6	132,4	110,5	115,3	148,8	119,4	116,5
14	131,7	162,3	137,1	156,2	148,8	133,5	135,5	105,8	119,0	146,9	124,7	117,0
15	130,5	161,3	138,1	158,8	149,4	134,5	130,3	99,6	119,8	142,4	126,7	115,0
16	131,6	159,6	141,1	157,4	149,6	135,6	133,9	98,8	119,7	141,4	124,7	113,2
