# Supplementary material for: Cytokine response and damages in the lungs of aging Syrian hamsters on a high-fat diet infected with the SARS-CoV-2 virus
Source: Front Immunol. 2023 Jul 14;14:1223086. doi: 10.3389/fimmu.2023.1223086 (PMC10375707; doi:10.3389/fimmu.2023.1223086)
Supplement: Supplementary file 1 [file DataSheet_1.zip › S 3 Table..pdf]

**S 3 Table. Biochemical parameters of blood serum of Syrian hamsters from different age groups**

| | | | | | |
|---|---|---|---|---|---|
| **Cholesterol (mg/dl)** | | | | | |
| Males (14 month) | 3,05 | 2,85 | 4,61 | 3,7 | 2,6 |
| Females (14 month) | 3,21 | 2,11 | 2,37 | 2,5 | 2,4 |
| Males (18 month) | 9,2 | 8,3 | 8,5 | 9,4 | 8,7 |
| Females (18 month) | 8,9 | 9,7 | 7,3 | 9,5 | 8,6 |
| **Triglycerides (mg/dl)** | | | | | |
| Males (14 month) | 2,56 | 1,08 | 2,37 | 1,03 | 1,19 |
| Females (14 month) | 5,11 | 2,32 | 2,03 | 2,50 | 3,40 |
| Males (18 month) | 11,2 | 9,8 | 10,8 | 8,9 | 10,3 |
| Females (18 month) | 9,5 | 10,2 | 8,8 | 10,9 | 11,1 |
| **Glucose (mg/dl)** | | | | | |
| Males (14 month) | 6,94 | 13,05 | 11,78 | 9,7 | 10,3 |
| Females (14 month) | 5,94 | 9,44 | 9,77 | 6,8 | 7,9 |
| Males (18 month) | 7,6 | 8,7 | 5,9 | 6,2 | 10,9 |
| Females (18 month) | 5,9 | 9,2 | 10,8 | 6,5 | 7,7 |
| **Aspartate aminotransferase (U/l)** | | | | | |
| Males (14 month) | 44,88 | 36,27 | 58,03 | * | * |
| Females (14 month) | 70,27 | 40,35 | 77,97 | * | * |
| Males (18 month) | 48,95 | 65,0 | 73,58 | 79,12 | 59,01 |
| Females (18 month) | 62,3 | 71,82 | 51,00 | 75,71 | 83,40 |
| **Alanine aminotransferase (U/l)** | | | | | |
| Males (14 month) | 74,57 | 48,86 | 38,0 | * | * |
| Females (14 month) | 87,71 | 80,86 | 66,0 | * | * |
| Males (18 month) | 80,2 | 58,85 | 72,23 | 64,0 | 85,64 |
| Females (18 month) | 68,32 | 79,25 | 71,9 | 85,64 | 55,05 |
| * small sample | | | | | |
