# Supplementary material for: Cytokine response and damages in the lungs of aging Syrian hamsters on a high-fat diet infected with the SARS-CoV-2 virus
Source: Front Immunol. 2023 Jul 14;14:1223086. doi: 10.3389/fimmu.2023.1223086 (PMC10375707; doi:10.3389/fimmu.2023.1223086)
Supplement: Supplementary file 1 [file DataSheet_1.zip › S 4 Table..pdf]

S 4 Table. Weight dynamics of Syrian hamsters after infection with the SARS-COV-2 virus

**Males on RD diet (g)**						
0 d.p.i.	133,3	124,0	130,6	146,8	137,2	*
3 d.p.i	125,9	110,8	118,4	129,0	135,2	*
**Males on HF diet (g)**						
0 d.p.i.	119,7	106,2	126,4	116,8	80,0	127,0
3 d.p.i	108,9	98,3	115,0	105,0	78,3	112,3
**Females on RD diet (g)**						
0 d.p.i.	120,4	140,0	121,5	134,0	118,8	192,2
3 d.p.i	109,1	127,5	110,9	125,3	120,9	172,0
**Females on HF diet (g)**						
0 d.p.i.	123,8	126,9	127,4	144,4	123,4	108,9
3 d.p.i	116,6	112,8	118,2	129,9	122,8	102,3
* died under anesthesia						
