# Supplementary material for: Cytokine response and damages in the lungs of aging Syrian hamsters on a high-fat diet infected with the SARS-CoV-2 virus
Source: Front Immunol. 2023 Jul 14;14:1223086. doi: 10.3389/fimmu.2023.1223086 (PMC10375707; doi:10.3389/fimmu.2023.1223086)
Supplement: Supplementary file 1 [file DataSheet_1.zip › S 5 Table..pdf]

S 5 Table. Cytokine levels in Syrian hamster's lung homogenates

TNF-α, Male (pg/ml)										
**Groups**	RD Diet					HF diet				
Health	1164,7	1605,3	1531,6	1694,1	760,7	1670,3	1909,2	1663,1	1431,6	1689,5
SARS-CoV-2	1423,3	720,4	1264,6	1281,7	1172,0	824,7	1780,1	1302,35	1541,1	1362,1
**TNF-α, Female (pg/ml)**										
**Groups**	RD Diet					HF diet				
Health	1343,9	2692,7	1820,1	1445,3	1799,5	2060,8	1971,5	1298,5	1230,0	870,4
SARS-CoV-2	1056,6	1587,2	857,0	1015,9	764,5	1140,1	1472,2	1831,7	1652,0	850,0
**IL-6, Male (pg/ml)**										
**Groups**	RD Diet					HF diet				
Health	191,3	89,3	656,0	267,0	615,9	652,3	431,1	1547,6	647,6	890,9
SARS-CoV-2	1417,4	3608,4	5020,3	4622,0	3376,9	4980,4	2287,0	1832,7	3475,8	4803,6
**IL-6, Female (pg/ml)**										
**Groups**	RD Diet					HF Diet				
Health	381,4	1512,7	461,9	1328,2	215,8	1410,1	3504,6	2234,2	2150,7	1992,7
SARS-CoV-2	656,1	2174,6	2165,2	4797,6	3627,7	3297,7	2482,2	4113,2	2890,1	3195,7
**IFN-γ, Male (pg/ml)**										
**Groups**	RD Diet					HF Diet				
Health	105,1	155,1	117,3	157,9	133,85	164,6	179,1	222,8	213,3	179,9
SARS-CoV-2	170,8	113,9	205,2	222,0	142,1	169,9	201,5	185,9	128,8	205,4
**IFN-γ, Female (pg/ml)**										
**Groups**	RD Diet					HF Diet				
Health	110,1	115,0	134,5	124,9	170,6	187,0	260,0	175,5	139,0	103,9
SARS-CoV-2	249,3	159,0	235,5	81,6	104,8	206,5	241,6	113,2	197,2	246,3
**IFN-α, Male (pg/ml)**										
**Groups**	RD Diet					HF Diet				
Health	69,1	98,7	79,6	88,0	104,4	143,6	140,3	184,0	105,3	205,3
SARS-CoV-2	225,0	217,3	179,2	211,6	118,4	187,2	165,2	188,8	165,8	235,1
**IFN-α, Female (pg/ml)**										
**Groups**	RD Diet					HF Diet				
Health	91,4	123,6	136,6	121,5	120,6	107,5	285,4	157,7	128,7	137,9
SARS-CoV-2	196,6	163,5	223,7	77,3	156,3	190,0	216,8	138,0	196,0	164,4
