# Supplementary material for: Cytokine response and damages in the lungs of aging Syrian hamsters on a high-fat diet infected with the SARS-CoV-2 virus
Source: Front Immunol. 2023 Jul 14;14:1223086. doi: 10.3389/fimmu.2023.1223086 (PMC10375707; doi:10.3389/fimmu.2023.1223086)
Supplement: Supplementary file 1 [file DataSheet_1.zip › S 6 Table..pdf]

Table 6. Histological scores

Males				
**№**	**Health animals**		**Animals infected with SARS-CoV-2**	
	RD	HF	RD	HF
1	0	0	2	2
2	0	0	2	3
3	0	0	2	2
4	0	0	3	2
5	0	0	2	2
6	0	0	2	3
**Females**				
**№**	**Health animals**		**Animals infected with SARS-CoV-2**	
	RD	HF	RD	HF
1	0	0	2	1
2	0	0	1	3
3	0	0	2	2
4	0	0	2	2
5	0	0	1	2
6	0	0	3	2
